# Five years of change in adult twins: longitudinal changes of genetic and environmental influence on epigenetic clocks

**DOI:** 10.1186/s12916-024-03511-y

**Published:** 2024-07-10

**Authors:** Ke Miao, Shunkai Liu, Weihua Cao, Jun Lv, Canqing Yu, Tao Huang, Dianjianyi Sun, Chunxiao Liao, Yuanjie Pang, Runhua Hu, Zengchang Pang, Min Yu, Hua Wang, Xianping Wu, Yu Liu, Wenjing Gao, Liming Li

**Affiliations:** 1 Department of Epidemiology and Biostatistics, School of Public Health, Peking University, Beijing, 100191 China; 2 Key Laboratory of Epidemiology of Major Diseases, (Peking University), Ministry of Education, Beijing, 100191 China; 3 Qingdao Center for Disease Control and Prevention, Qingdao, China; 4 Zhejiang Center for Disease Control and Prevention, Hangzhou, China; 5 Jiangsu Center for Disease Control and Prevention, Nanjing, China; 6 Sichuan Center for Disease Control and Prevention, Chengdu, China; 7 Heilongjiang Center for Disease Control and Prevention, Harbin, China

**Keywords:** Epigenetic clock, Aging, Twin, Heritability

## Abstract

**Background:**

Epigenetic clocks were known as promising biomarkers of aging, including original clocks trained by individual CpG sites and principal component (PC) clocks trained by PCs of CpG sites. The effects of genetic and environmental factors on epigenetic clocks are still unclear, especially for PC clocks.

**Methods:**

We constructed univariate twin models in 477 same-sex twin pairs from the Chinese National Twin Registry (CNTR) to estimate the heritability of five epigenetic clocks (GrimAge, PhenoAge, DunedinPACE, PCGrimAge, and PCPhenoAge). Besides, we investigated the longitudinal changes of genetic and environmental influences on epigenetic clocks across 5 years in 134 same-sex twin pairs.

**Results:**

Heritability of epigenetic clocks ranged from 0.45 to 0.70, and those for PC clocks were higher than those for original clocks. For five epigenetic clocks, the longitudinal stability was moderate to high and was largely due to genetic effects. The genetic correlations between baseline and follow-up epigenetic clocks were moderate to high. Special unique environmental factors emerged both at baseline and at follow-up. PC clocks showed higher longitudinal stability and unique environmental correlations than original clocks.

**Conclusions:**

For five epigenetic clocks, they have the potential to identify aging interventions. High longitudinal stability is mainly due to genetic factors, and changes of epigenetic clocks over time are primarily due to changes in unique environmental factors. Given the disparities in genetic and environmental factors as well as longitudinal stability between PC and original clocks, the results of studies with original clocks need to be further verified with PC clocks.

**Supplementary Information:**

The online version contains supplementary material available at 10.1186/s12916-024-03511-y.

## Background

The process of aging is complex, and individuals of the same chronological age usually are on different aging trajectories [1]. Owing to the association between DNA methylation and aging, DNA methylation-based biomarkers of aging have been developed, called epigenetic clocks. Three compelling epigenetic clocks (GrimAge, PhenoAge, and DunedinPACE) have attracted widespread attention [2].

Epigenetic clocks were trained on age-associated molecular and physiological measures using machine learning [3–5]. The GrimAge clock was based on 1030 cytosine-phosphate-guanine (CpG) sites associated with 7 plasma proteins and smoking and had an excellent prediction of lifespan and healthspan [3]. The PhenoAge clock was developed by identifying 513 CpG sites derived from chronological age and 9 blood measures [5]. The DunedinPACE, based on a set of 173 CpG sites, was developed by using the longitudinal change rate of 19 biological measures over 20 years as training phenotypes [4]. Because of various training phenotypes, epigenetic clocks capture different aspects of aging and fall into two categories: (1) represent the progress of aging (GrimAge and PhenoAge) and (2) represent the pace of aging (DunedinPACE) [2].

At present, the methylation level of most individuals is detected by beadchip, and there is great technical noise from individual CpG sites [6]. Technical noise threatens the repeatability of epigenetic clocks [7]. Hence, the proposed principal component (PC) version of epigenetic clocks, called PC clocks (PCGrimAge and PCPhenoAge), have been trained by PCs of clock-related CpG sites to reduce technical noise and have shown more reliability in longitudinal studies [8].

Aging process differs among individuals, as it is driven by genetic and environmental factors [9, 10]. Based on DNA methylation information, epigenetic clocks are also influenced by both genetic and environmental factors. On the one hand, it has been proved in previous studies that epigenetic clocks are associated with lifestyle, BMI, and socioeconomic status [11, 12]. On the other hand, there are twin studies using a cross-sectional design to obtain the heritability of original clocks (GrimAge, PhenoAge, and DunedinPACE) [13–15], but the heritability of PC clocks remains unknown. Only one longitudinal twin study explored the longitudinal changes of the PhenoAge clock and found that the genetic factors of the PhenoAge clock at two measured times overlapped [16]. Longitudinal twin study provides a valuable design for examining changes in heritability and assessing the genetic and environmental influence on the longitudinal stability of epigenetic clocks over time [17]. Such a design also allows for assessing the overlap of genes and environmental effects in the epigenetic clocks over time [18]. Given the complexity of aging, we can use the longitudinal twin study and epigenetic clocks as aging biomarkers to explore the continuity of genetic factors at different stages of aging, as well as the relationship between genetic and environmental factors in the progression of aging.

Currently, to our knowledge, no studies have explored the heritability of PC clocks and the difference in heritability between original and PC clocks. Only one relevant study focused on the longitudinal changes in the genetic and environmental effects on the PhenoAge clock. Therefore, based on twins from the Chinese National Twin Registry (CNTR), we first explored the heritability of original and PC clocks in Chinese populations. Then, we conducted a longitudinal design to estimate the longitudinal changes of genetic and environmental effects on original and PC clocks, to explore the reasons for the discrepancy between original and PC clocks.

## Methods

### Study populations

The twins were assessed in two thematic surveys in 2013 and 2017–2018 within the CNTR [19]. For baseline and follow-up surveys, twins completed questionnaires and provided fasting blood samples after informed consent. A total of 477 same-sex twin pairs (954 twins) were included in the present study who had questionnaires and DNA methylation information in only one survey. Among them, 134 same-sex twin pairs (268 twins) had information in both baseline and follow-up surveys. Zygosity diagnosis of twins was conducted using a set of 59 SNPs based on DNA methylation information [20]. Ethical approval was obtained from the Biomedical Ethics Committee at Peking University (IRB00001052-13022, IRB00001052-14021).

### DNA methylation profiling

Genomic DNA was extracted from fasting peripheral blood samples. Of 954 twins who participated in only one survey, 253 twins used the Illumina Human Methylation 450 K BeadChip array and 701 twins used the Illumina Infinium Methylation EPIC (850 K) BeadChip array to obtain DNA methylation data. For 268 twins who participated in both two surveys, the 450 K array was employed on 107 twins for the baseline survey, and the 850 K array was employed on 161 twins for the baseline survey and on 268 twins for the follow-up survey. We used the R package minfi (1.40.0) to combine the methylation data from different arrays and then preprocess [21]. Only overlapped probes on both 450 K and 850 K arrays were retained for subsequent analyses, using the “combineArrays” function. We compared each probe with the background signal level to calculate detection *P* values by using the “detection” function. Bad-quality probes (detection *P* values > 0.05 in more than 1% of samples) and samples of poor quality (detection *P* values > 0.01) were removed from further analysis. We also excluded probes that were multi-hit probes, were SNP-related, or were located in chromosome X and Y. We used the quantile normalization method to normalize methylation data and used β-value (number of methylated probes/[number of both methylated and unmethylated probes]) to represent the DNA methylation level. We used the “ComBat” function of R package sva (3.42.0) to adjust for known batch effects within the DNA methylation data [22]. Finally, 409,303 CpG sites remained for the calculation of epigenetic clocks.

### Epigenetic clocks

We calculated five epigenetic clocks. The first two clocks, GrimAge and PhenoAge, are composite measures of 1030 [3] and 513 [5] CpG sites, respectively. GrimAge and PhenoAge were assessed using the online calculator (http://dnamage.genetics.ucla.edu/) [23]. Beta values were used as input, and the normalization method implemented in the calculator was utilized. These two epigenetic clocks were calculated by aggregating the weighted averages of β-values at specific CpG sites. For the unreliability of individual CpG sites, we also calculated PC-based GrimAge and PhenoAge (“PCGrimAge” and “PCPhenoAge”), which constructed principal component analysis (PCA) on the DNA methylation data to extract covariance among multicollinear CpG sites [8]. The PCs consist of multiple CpG sites, and technical noise is unlikely to covary with age-related signals [8]. Thus, the PCs of CpG sites captured the majority of age-related signals while minimizing the influence of technical noise. PC clocks were obtained by using PCs to retrain the original clocks. PC clocks were calculated using the R code given by the researchers (https://github.com/MorganLevineLab/PC-Clocks/) [8]. For these epigenetic clocks, age acceleration (AA) was defined as the residuals obtained from regressing the epigenetic clocks on six blood cell components and chronological age (GrimAA, PhenoAA, PCGrimAA, and PCPhenoAA, respectively). We used the Houseman method to assess the six blood cell components, including CD4^+^ T cells, CD8^+^ T cells, B cells, monocytes, granulocytes, and natural killer cells [24]. AA indicated the degree of aging compared to the chronological age. The fifth clock was DunedinPACE, which represented the pace of biological aging [4]. DunedinPACE was calculated by using the public R package “DunedinPACE” (https://github.com/danbelsky/DunedinPACE). DunedinPACE removes unreliable CpG sites during the training process, so it does not have a PC version. Four measures of AA and DunedinPACE were epigenetic age metrics. To make the results comparable, we normalized epigenetic age metrics before inputting in hereafter twin modeling.

### Statistical analyses

#### Univariate twin models

First, we calculated the heritability of five epigenetic age metrics in 954 twins using univariate structural equation modelling (SEM). The variance of epigenetic age metrics was decomposed into four latent variables, including additive genetic (A), common environmental (C), or dominant genetic (D) and unique environmental (E) components [18]. A represents the cumulative impact of individual alleles at various loci affecting epigenetic age metrics. C represents the common environment twins share. D represents the interplay among alleles at the same locus or different loci. E represents the different environment among twins and measurement error. Monozygotic twins (MZ) share 100% of their genes (all additive and dominant effects), because they developed from the same fertilised egg. Dizygotic twins (DZ) share on average 50% of their segregating genes, that is 25% for the dominant effects and 50% for the additive effects [25]. The design with twins reared together cannot provide sufficient information to estimate both C and D, so we can only conduct either an ACE or an ADE model [26]. For the ACE model, the heritability of the epigenetic age metric is the proportion of the variance of A to the total variance; for the ADE model, the heritability is the proportion of the variance of A and D to the total variance. Then, A, C or D were dropped respectively to fit sub-models of ACE or ADE. We conducted a comparative analysis between the ACE or ADE model and the fully-saturated model, as well as between the sub-models and the ACE or ADE model, using the likelihood-ratio test (LRT). The not statistically significant (*P*>0.05) result of LRT indicates that the more parsimonious model fits the data as well as the more complex model. Besides, we used Akaike's information criterion (AIC) value to measure model parsimony: the smaller the AIC, the more parsimonious the fit [27]. We chose the most parsimonious model that fits the data as the optimal model.

#### Longitudinal bivariate twin models

**Fig. 1:**
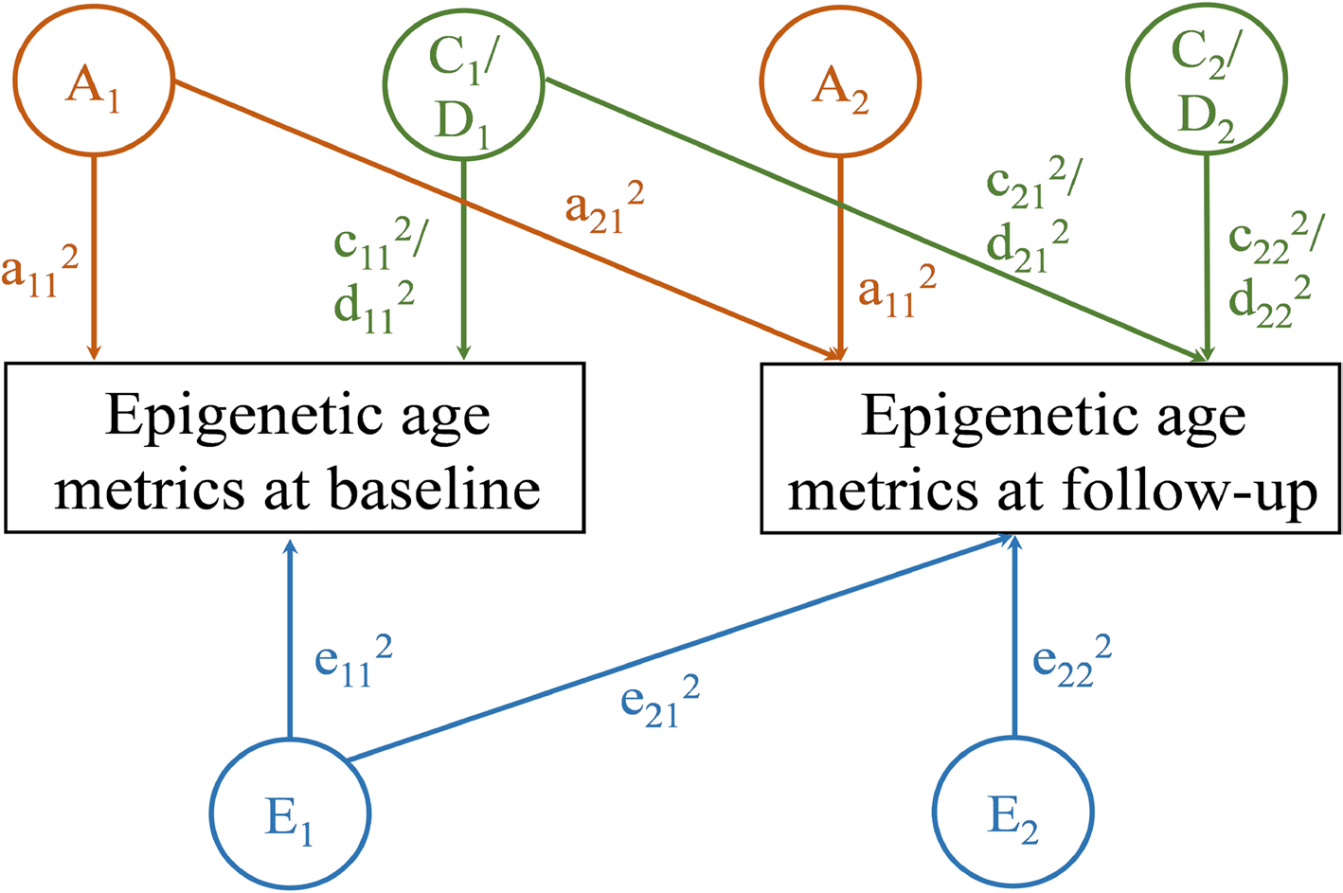
Bivariate Cholesky model. Note. Components of variance: a_11_^2^, the variance of A at baseline; c_11_^2^, the variance of C at baseline; d_11_^2^, the variance of D at baseline; e_11_^2^, the variance of E at baseline; a_22_^2^, the variance of A unique at follow-up; c_22_^2^, the variance of C unique at follow-up; d_22_^2^, the variance of D unique at follow-up; e_22_^2^, the variance of E unique at follow-up. Components of covariance: a_21_^2^, the covariance of A between baseline and follow-up; c_21_^2^, the covariance of C between baseline and follow-up; d_21_^2^, the covariance of D between baseline and follow-up; e_21_^2^, the covariance of E between baseline and follow-up. Orange color indicates the effect of A; green color indicates the effect of C or D; blue color indicates the effect of E

We used bivariate Cholesky twin models to explore the longitudinal changes of the genetic and environmental influence on epigenetic age metrics in two surveys [17]. For the baseline survey, the variance of epigenetic age metrics was decomposed into A (a_11_^2^), C (c_11_^2^) or D (d_11_^2^), and E (e_11_^2^), respectively. For the follow-up survey, the variance of epigenetic age metrics was decomposed into A (a_22_^2^), C (c_22_^2^) or D (d_22_^2^), and E (e_22_^2^), which were all independent of genetic and environmental effects at baseline. The bivariate ACE/ADE model also decomposes the covariance of epigenetic age metrics across time into A, C or D, and E components as a_21_^2^, c_21_^2^ or d_21_^2^, and e_21_^2^ (Fig. 1). For the ACE model, the heritability at baseline was given by a_11_^2^/(a_11_^2^ + c_11_^2^ + e_11_^2^), whereas the heritability at follow-up was given by (a_21_^2^ + a_22_^2^)/(a_21_^2^ + a_22_^2^ + c_21_^2^ + c_22_^2^ + e_21_^2^ + e_22_^2^). Moreover, we calculated the cross-twin cross-trait (CTCT) correlations for MZ and DZ twins, which describe the correlation between twin 1’s epigenetic age metrics at baseline and the co-twin’s epigenetic age metrics at follow-up. If the CTCT for MZ twins is higher than that for DZ twins, genetic factors affect the change of epigenetic age metrics across time. We calculated the phenotypic correlation (R_ph_) of epigenetic age metrics across times and decomposed R_ph_ into common genetic correlation (R_a_), common environmental correlation (R_c_), and unique environmental correlation (R_e_) [18]. Since it is the same variable across time, we used the phenotype correlation to represent the longitudinal stability of epigenetic age metrics. The proportion of the R_ph_ can be derived from genetic (P_a_), common environmental (P_c_), and unique environmental (P_e_) parts [18]. The model selection criteria is as described above.

Twin models were performed using the *OpenMx* (version 2.20.6) package, version R 4.1.2. Because AA was the residual from epigenetic clocks regression to chronological age, the models of four AAs were adjusted for only sex, and the models for DunedinPACE were adjusted for sex and chronological age. *P* < 0.05 indicated statistical significance.

## Results

### Descriptive statistics

Among the 954 (477 pairs) adult twins included in the cross-sectional study, 79.7% were MZ twins; 70.2% were males; and the mean (standard deviation [SD]) age was 50.30 (11.86) years (range 19.00 ~ 82.00 years). The means (SD) of original and PC clocks were as follows: GrimAge 50.30 (10.44) years, PCGrimAge 65.46 (9.79) years, PhenoAge 47.65 (9.79) years, PCPhenoAge 56.10 (10.09) years, and DunedinPACE 1.14 (0.12) units. The longitudinal analysis included 268 (134 pairs) twins who participated in baseline and follow-up surveys, with means age of 50.24 (range 26.00 ~ 77.00) years at baseline and 54.87 (range 31.00 ~ 82.00) years at follow-up (Table 1).

**Table 1:** Characteristics of the study population

Cross-sectional study		Longitudinal study	
		Baseline	Follow-up
*N*	954	268	268
Chronological age	50.30 ± 11.86	50.24 ± 10.27	54.87 ± 10.21
Age group			
≤ 25 years	22 (2.3)	0 (0.0)	0 (0.0)
25 ~ 45 years	282 (29.6)	84 (31.4)	48 (17.9)
45–60 years	458 (48.0)	140 (52.2)	142 (53.0)
> 60 years	192 (20.1)	44 (16.4)	78 (29.1)
MZ pairs (%)	380 (79.7)	95 (70.9)	
DZ pairs (%)	97 (20.3)	39 (29.1)	
Male (%)	670 (70.2)	168 (62.7)	
GrimAge	50.30 ± 10.44	49.45 ± 9.08	54.00 ± 8.56
PCGrimAge	65.46 ± 9.79	65.00 ± 8.47	68.87 ± 8.31
PhenoAge	47.65 ± 9.79	49.13 ± 8.87	50.54 ± 9.23
PCPhenoAge	56.10 ± 10.09	58.16 ± 8.79	62.25 ± 8.28
^a^GrimAA	0.10 ± 3.76	− 0.03 ± 3.81	0.03 ± 3.56
^a^PCGrimAA	0.06 ± 2.42	0.00 ± 2.42	0.00 ± 2.37
^a^PhenoAA	− 0.08 ± 4.61	− 0.23 ± 5.05	− 0.30 ± 4.96
^a^PCPhenoAA	0.10 ± 3.98	− 0.22 ± 4.13	− 0.26 ± 3.52
DunedinPACE	1.14 ± 0.12	1.13 ± 0.10	1.14 ± 0.11

Data are presented as *n* (%) or mean ± standard deviation

*Abbreviations:* *MZ* monozygotic, *DZ* dizygotic

^a^Epigenetic age accelerations (AA) are derived from adjusting epigenetic age for blood cell counts and chronological age

The correlation between GrimAge and PCGrimAge (*r* = 0.98, *P* < 0.001) was higher than that between PhenoAge and PCPhenoAge (*r* = 0.88, *P* < 0.001) (Additional file 1: Fig. S1). The correlations between epigenetic clocks and chronological age ranged from 0.38 to 0.97. The highest was for PCGrimAge (*r* = 0.97), and the lowest was for DunedinPACE (*r* = 0.38). The correlations between PC clocks and chronological age exceeded that between original clocks and chronological age (Additional file 1: Fig. S2).

### Heritability of epigenetic age metrics

In univariate analyses, for all the five epigenetic age metrics, ACE models provided better fits to the data than ADE models (Additional file 2: Table S1). Then, we conducted sub-models of the ACE model. We found that the best-fit models for four epigenetic age metrics (GrimAA, PCGrimAA, PhenoAA, and PCPhenoAA) were the AE model, but that for DunedinPACE was the ACE model (Additional file 2: Table S1).

Table 2 shows the variance explained by three components (A, C, and E) and the heritability of five epigenetic age metrics. The heritability of GrimAA and PCGrimAA were 0.61 and 0.69, respectively, and no significant difference existed between them according to the overlapped 95% confidence interval (CI). The heritability of PhenoAA (0.45, 95% CI 0.38 ~ 0.53) was significantly lower than that of PCPhenoAA (0.70, 95% CI 0.65 ~ 0.75). The heritability of DunedinPACE was 0.59.

**Table 2:** Parameter estimates (95% CI) from the univariate model of epigenetic age metrics (*N* = 954)

Epigenetic age metrics	a^2^ (95% CI)	c^2^ (95% CI)	e^2^ (95% CI)	h^2^ (95% CI)	e^2^/total variance (95% CI)
GrimAA	0.48 (0.41, 0.57)	–	0.30 (0.26, 0.35)	0.61 (0.55, 0.67)	0.39 (0.33, 0.45)
PCGrimAA	0.52 (0.45, 0.61)	–	0.23 (0.20, 0.27)	0.69 (0.64, 0.74)	0.31 (0.26, 0.36)
PhenoAA	0.45 (0.35, 0.56)	–	0.54 (0.47, 0.62)	0.45 (0.38, 0.53)	0.55 (0.47, 0.62)
PCPhenoAA	0.69 (0.59, 0.81)	–	0.30 (0.26, 0.34)	0.70 (0.65, 0.75)	0.30 (0.25, 0.35)
DunedinPACE	0.52 (0.25, 0.61)	0.00 (0.00, 0.25)	0.35 (0.31, 0.41)	0.59 (0.29, 0.65)	0.41 (0.35, 0.47)

*a*^*2*^ variance explained by additive genetic component, *c*^*2*^ variance explained by common environmental component, *e*^*2*^ variance explained by unique environmental component, *h*^*2*^ heritability, that is, the ratio of a^2^ to the total variance

### Genetic and environmental evolution of epigenetic age metrics over time

The R_ph_ of epigenetic age metrics at baseline and follow-up ranged from 0.50 to 0.85, indicating the longitudinal stability was moderate to strong. The R_ph_ of PC clocks were higher than their corresponding original clocks. The CTCT correlations of epigenetic age metrics were all higher within MZ twin pairs than that within DZ twin pairs, suggesting the influence of genetic factors (Table 3). Four epigenetic age metrics (GrimAA, PCGrimAA, PhenoAA, and DunedinPACE) showed the optimal model was the AE model, while that for PCPhenoAA was the ACE model (Additional file 2: Table S2).

**Table 3:** The correlations from bivariate models of epigenetic age metrics (*N* = 268)

Epigenetic age metrics		CTCT r	R_ph_	R_a_	R_e_	P_a_	P_e_
GrimAA	MZ	0.50 (0.34, 0.64)	0.70 (0.62, 0.76)	0.85 (0.74, 0.94)	0.38 (0.21, 0.54)	86%	14%
	DZ	0.41 (0.11, 0.64)					
PCGrimAA	MZ	0.69 (0.56, 0.78)	0.85 (0.81, 0.88)	0.94 (0.89, 0.98)	0.61 (0.47, 0.72)	85%	15%
	DZ	0.17 (− 0.15, 0.46)					
PhenoAA	MZ	0.37 (0.18, 0.53)	0.50 (0.39, 0.59)	0.78 (0.56, 1.00)	0.22 (0.03, 0.40)	86%	14%
	DZ	0.19 (− 0.13, 0.48)					
PCPhenoAA	MZ	0.48 (0.31, 0.62)	0.60 (0.50, 0.68)	0.81 (0.58, 1.00)	0.67 (0.55, 0.77)	75%	25%
	DZ	0.07 (− 0.25, 0.38)					
DunedinPACE	MZ	0.60 (0.45, 0.71)	0.76 (0.70, 0.81)	0.99 (0.91, 1.00)	0.36 (0.20, 0.52)	91%	9%
	DZ	0.44 (0.15, 0.66)					

*CTCT r*, cross-twin cross-trait (CTCT) correlations; *R*_*ph*_, phenotypic correlation; *R*_*a*_, genetic correlation; *R*_*e*_, unique environmental correlation; *P*_*a*_, the proportion of R_ph_ due to genetic factors; *P*_*e*_, the proportion of R_ph_ due to unique environmental factors

**Fig. 2:**
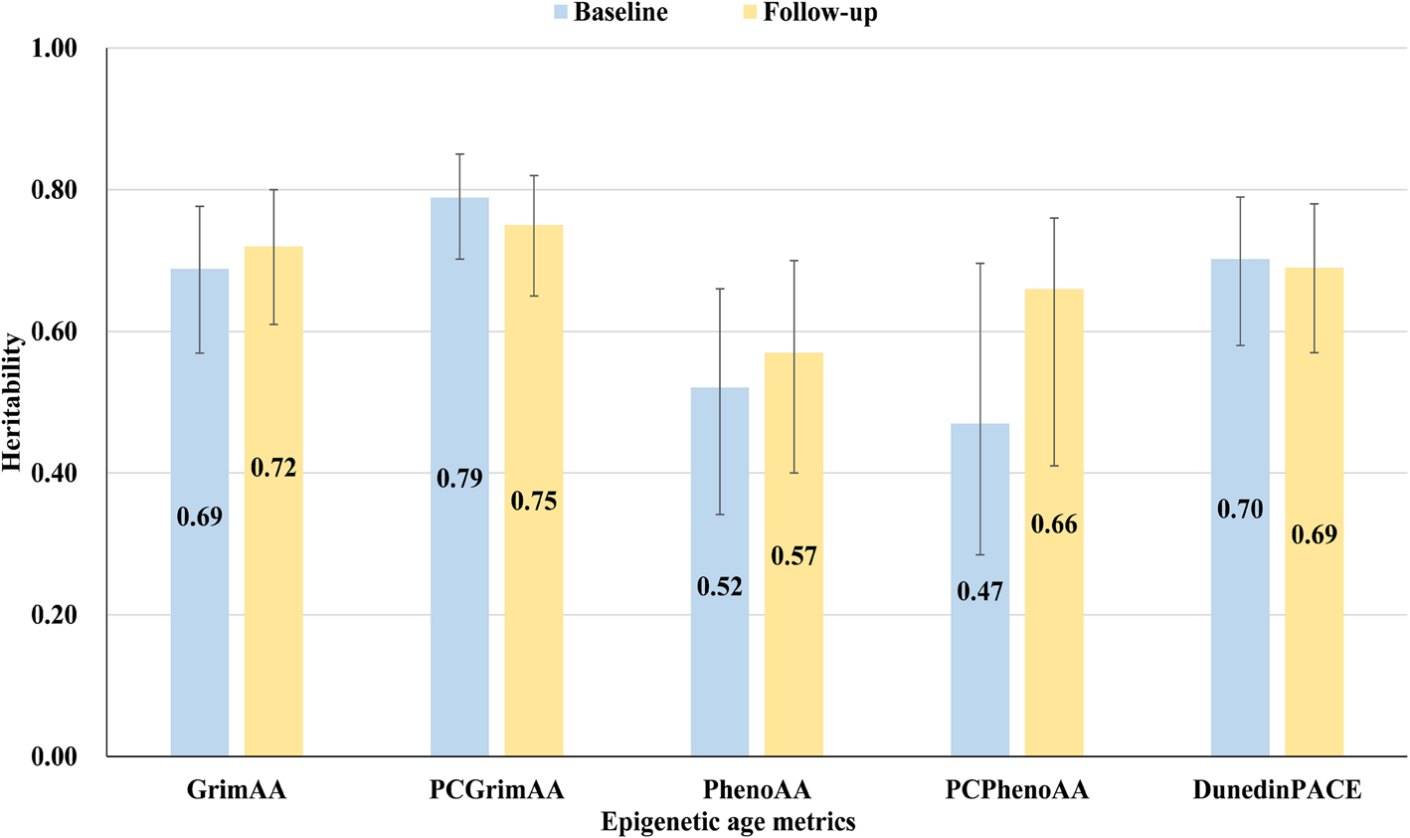
Heritability of epigenetic age metrics in bivariate twin models (*N* = 268)

Modest decreases were obtained in the heritability of PCGrimAA (from 0.79 to 0.75) and DunedinPACE (from 0.70 to 0.69) over time, but this trend was not obtained in GrimAA (from 0.69 to 0.72), PhenoAA (from 0.52 to 0.57), and PCPhenoAA (from 0.47 to 0.66) (Fig. 2, Additional file 2: Table S3).

**Fig. 3:**
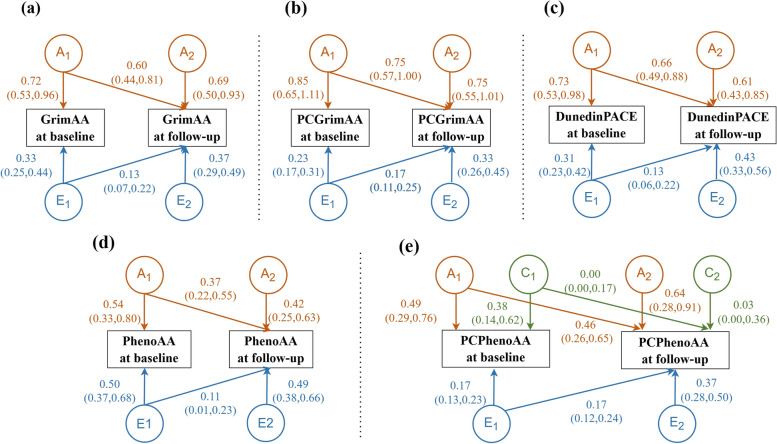
Bivariate twin models for epigenetic age metrics (*N* = 268). Note: Orange color indicates the effect of A; green color indicates the effect of C or D; blue color indicates the effect of E

Figure 3 shows the variance and covariance of three components (A, C, and E) for five epigenetic age metrics. For GrimAA, PCGrimAA, PhenoAA, and DunedinPACE, the specific variance of A at baseline (a_11_^2^) were higher than that specific at follow-up (a_22_^2^). However, for PCPhenoAA, a_22_^2^ was higher than a_11_^2^. The covariance of A between baseline and follow-up (a_21_^2^) were lower than or equal to a_22_^2^ for four epigenetic age metrics (not DunedinPACE). Except for PhenoAA, the specific variance of E at baseline (e_11_^2^) were lower than those specific at follow-up (e_22_^2^). The covariance of E (e_21_^2^) were all lower than e_22_^2^. For PCPhenoAA, the variance of C specific at follow-up (c_22_^2^) and the covariance of C (c_21_^2^) were close to zero, indicating that the role of common environmental factors declines over time.

The genetic correlations (R_a_) were all high, especially of PCGrimAA (R_a_ = 0.94) and DunedinPACE (R_a_ = 0.99). The unique environmental correlations (R_e_) were low to moderate (ranging from 0.22 to 0.67). PC clocks showed higher R_a_ and R_e_ than original clocks. For epigenetic age metrics, R_ph_ were largely due to genetic effects, with the proportion of R_ph_ due to genetic factors (P_a_) ranging from 75 to 91% (Table 3).

## Discussion

Overall, we conducted univariate twin models of epigenetic age metrics in 954 twins and conducted longitudinal bivariate twin models in 268 twins who participated in baseline and follow-up surveys. Results from univariate models showed the moderate to high heritability of epigenetic age metrics (range from 0.45 to 0.70). The results from longitudinal bivariate models showed genetic contributions to epigenetic age metrics decreased, and unique environmental contributions increased across time. There was a large overlap in genetic effects and a small overlap in unique environmental effects across 5 years. However, for all epigenetic clocks, new genetic and new unique environmental influences emerged across time. Moreover, the phenotypic correlation representing the longitudinal stability of epigenetic clocks were moderate to strong, mainly due to genetic factors. PC clocks showed higher correlations with chronological age, higher heritability, greater longitudinal stability, and higher genetic and unique environmental correlations than original clocks.

To our knowledge, a few studies have investigated the heritability of epigenetic clocks, and virtually no study has explored the heritability of PC clocks. Some studies used polygenic models to measure heritability of epigenetic age accelerations and obtained low to moderate SNP-based heritability estimates: GrimAA at 0.10 ~ 0.30 [3, 28] and PhenoAA at 0.10 ~ 0.33 [5, 28]. However, similar to our results, other twin studies have reported the moderate to high heritability of epigenetic clocks [13–15]. A twin study estimated the heritability of GrimAA at 0.58 (95% CI 0.51 ~ 0.65) involving 1424 twins on average 34.5 years (age ranged 21 ~ 73 years) [13]. A study calculated the heritability in two twin cohorts and found that the heritability of GrimAA and PhenoAA in the young cohort (age ranged 21 ~ 25 years) were 0.62 and 0.64, and that in the older cohort (age ranged 55 ~ 72 years) were 0.58 and 0.60, respectively [14]. Only one study reported on the heritability of DunedinPACE at 0.68 (95% CI 0.52 ~ 0.82), which involved 730 twins with a mean age of 22.4 years [15]. It also estimated the heritability of GrimAA at 0.73 (95% CI 0.66 ~ 0.80) and PhenoAA at 0.65 (95% CI 0.56 ~ 0.74) [15]. All three studies involved twins from the Finnish Twin Cohort, fitted the AE model for epigenetic clocks and had a higher estimation of the heritability of epigenetic clocks (GrimAA, PhenoAA, and DunedinPACE) than our results. This may be due to the older age of our participants (age ranged 19 ~ 82 years, mean age of 50.3 years). From the longitudinal bivariate models, we proved that the estimate of heritability for DunedinPACE decreased over time. We utilized the cross-sectional design (954 twins) to obtain the heritability in the Chinese population. Besides, we used a longitudinal design (268 twins) to understand changes of the heritability.

Even so, new genetic factors still emerged at follow-up. Moreover, the genetic correlations of epigenetic clocks were high, and it could either be due to a large overlap in genes across time or due to persistent effects of genetic factors at baseline [16]. Given the emerging genetic factors at follow-up, we advocated the latter assumption. The genetic components for epigenetic clocks across time appeared pleiotropic, indicating the mechanisms of genes behind aging are complex. Our study confirms that age affects gene expression and that the influence of early genetic factors can extend into later periods. A previous longitudinal twin study investigated the PhenoAge clock and thought that the high genetic correlation of PhenoAge was attributed to the large overlap of genes, because no new genetic effects emerged during the follow-up period [16]. The discrepancy in outcomes may be attributed to the fact that the participants in the previous study had a mean age of 69.8 years at baseline and 78.9 years at follow-up, which is older than our study participants. Age significantly impacts gene expression, and the effect is more pronounced in older adults [29].

Contributions of unique environmental factors increased over time. New unique environmental factors emerged at follow-up, consistent with the previous study [16], indicating that epigenetic clocks would still be responsive to environmental factors even at old age, which further reveals the potential of epigenetic clocks as biomarkers of aging for the identification and evaluation of longevity interventions. However, we found significant unique environmental correlation across time, which were not found in the previous study [16]. The length of the follow-up period may be the reason for the discrepancy of unique environmental correlation between the two studies. Epigenetic clocks showed moderate to high longitudinal stability, most of which was due to genetic factors, and changes of epigenetic clocks over time were mainly due to changes in unique environmental factors, consistent with previous longitudinal twin studies for clock-related CpG sites and the PhenoAge clock [16, 17].

The difference between original and PC clocks lies in CpG sites. The former was trained from individual CpG sites, and the latter was trained from PCs of CpG sites to minimize the technical noise [8]. PCA was used to extract information from higher dimensions (several possibly correlated variables, as individual CpG sites) to lower dimensions (a smaller number of uncorrelated variables, as PCs) [30]. PC clocks discarded the low-variance PCs that primarily represent noise or otherwise do not contribute to prediction [31]. Notably, PC clocks were more strongly correlated with chronological age because the information of chronological age was contained in the PCs with a large variance, which was retained in PC clocks [8]. As expected, we found that PC clocks showed higher longitudinal stability than original clocks. The higher longitudinal stability of PC clocks was mainly due to removing technical noise which is expected to belong to the unique environmental component. We also found that the heritability and unique environmental correlation of PCPhenoAA were significantly higher than that of PhenoAA. The removal of technical noise allowed the preservation of truly unique environmental factors and increased the proportion of genetic factors. As demonstrated in previous studies [8, 32], compared to PCGrimAge and GrimAge, the correlation between PCPhenoAge and PhenoAge was weaker. This may be due to the fact that PCPhenoAge chose a smaller proportion of principal components (PCPhenoAge: 1000 of 4504 PhenoAge PCs; PCGrimAge: 1936 of 3934 GrimAge PCs) in training.

As far as we know, only one study has applied both original and PC clocks to examine the degree of aging [33]. Only a few longitudinal studies used PC clocks to evaluate the influence factors or interventions of aging [32, 34–38]. Because of the different study designs and populations, it is difficult to compare results from PC clocks with those from original clocks. However, due to the differences between PC and original clocks in genetic and environmental effects and longitudinal stability, the results of previous studies using original clocks, especially longitudinal studies, should be verified with PC clocks. Future longitudinal studies also need to take into account PC clocks instead of original clocks as biomarkers of aging.

The strengths of our study include the longitudinal design to investigate genetic and environmental effects behind the changes of epigenetic clocks over time, and the comparison between original and PC clocks. Nevertheless, some limitations should be considered. First, we relied on confidence intervals to judge the difference of genetic and environmental effects between original and PC clocks. When confidence intervals overlapped, we could not clearly judge whether there was a significant difference. In addition, given the limited sample size, further analysis cannot be taken into consideration, such as using the sex-limitation model to explore the effect of sex. Finally, the structural equation model can only approximate the impact of genetic and environmental factors on epigenetic clocks, and the specific genetic mechanism and environmental factors still need to be explored in future studies.

## Conclusions

In conclusion, we found the moderate to high heritability of epigenetic clocks and explored the extent to which the effects of genetic and environmental factors contribute to epigenetic clocks across 5 years. High longitudinal stability is mainly due to genetic factors, and changes of epigenetic clocks over time are mainly due to changes in unique environmental factors. The effects of genetic factors at baseline are persistent, and the mechanisms of genes behind epigenetic clocks are complex. The potential of epigenetic clocks as markers of aging for identifying interventions affecting aging was demonstrated, even at old age. PC clocks showed higher heritability and longitudinal stability than original clocks due to the reduction in technical noise belonging to unique environmental factors. Due to the differences between PC and original clocks in the genetic and environmental effects and longitudinal stability, the results of studies with the original clocks need to be further verified with PC clocks. Further studies should focus on PC clocks to explore the aging mechanism.

### Supplementary Information

Additional file 1: Figs. S1-S2. Fig.S1- [Correlations between the original clocks and their PC clock proxies in the overall samples]. Fig.S2- [Matrix heatmap of epigenetic age and chronological age].

Additional file 2: Table S1-S3. Table.S1- [Univariate model fit comparisons for the epigenetic age metrics in the cross-sectional analysis]. Table.S2- [Bivariate Model fit comparisons for the epigenetic age metrics in longitudinal analysis]. Table.S3- [Parameter estimates (95%CI) from bivariate twin models of epigenetic age metrics (*N*=268)].

## Data Availability

The datasets generated and analysed during the current study are not publicly available but are available from the corresponding author upon reasonable request.

## Abbreviations

A: Additive genetic component
AA: Age acceleration
AIC: Akaike’s information criterion
C: Common environmental component
CI: Confidence interval
CNTR: Chinese National Twin Registry
CpG: Cytosine-phosphate-guanine
CTCT: Cross-twin cross-trait
D: Dominant genetic component
DZ: Dizygotic twin
E: Unique environmental component
LRT: Likelihood-ratio test
MZ: Monozygotic twin
P_a_: The proportion of R_ph_ due to genetic factors
P_c_: The proportion of R_ph_ due to common environmental factors
PC: Principal component
PCA: Principal component analysis
P_e_: The proportion of R_ph_ due to unique environmental factors
R_a_: Common genetic correlation
R_c_: Common environmental correlation
R_e_: Unique environmental correlation
R_ph_: Phenotypic correlation
SD: Standard deviation
SEM: Structural equation modeling
