# Supplementary material for: Five years of change in adult twins: longitudinal changes of genetic and environmental influence on epigenetic clocks
Source: BMC Med. 2024 Jul 10;22:289. doi: 10.1186/s12916-024-03511-y (PMC11234599; doi:10.1186/s12916-024-03511-y)
Supplement: Supplementary file 1 — Additional file 1: Figs. S1-S2. Fig.S1- [Correlations between the original clocks and their PC clock proxies in the overall samples]. Fig.S2- [Matrix heatmap of epigenetic age and chronological age]. [file 12916_2024_3511_MOESM1_ESM.docx]

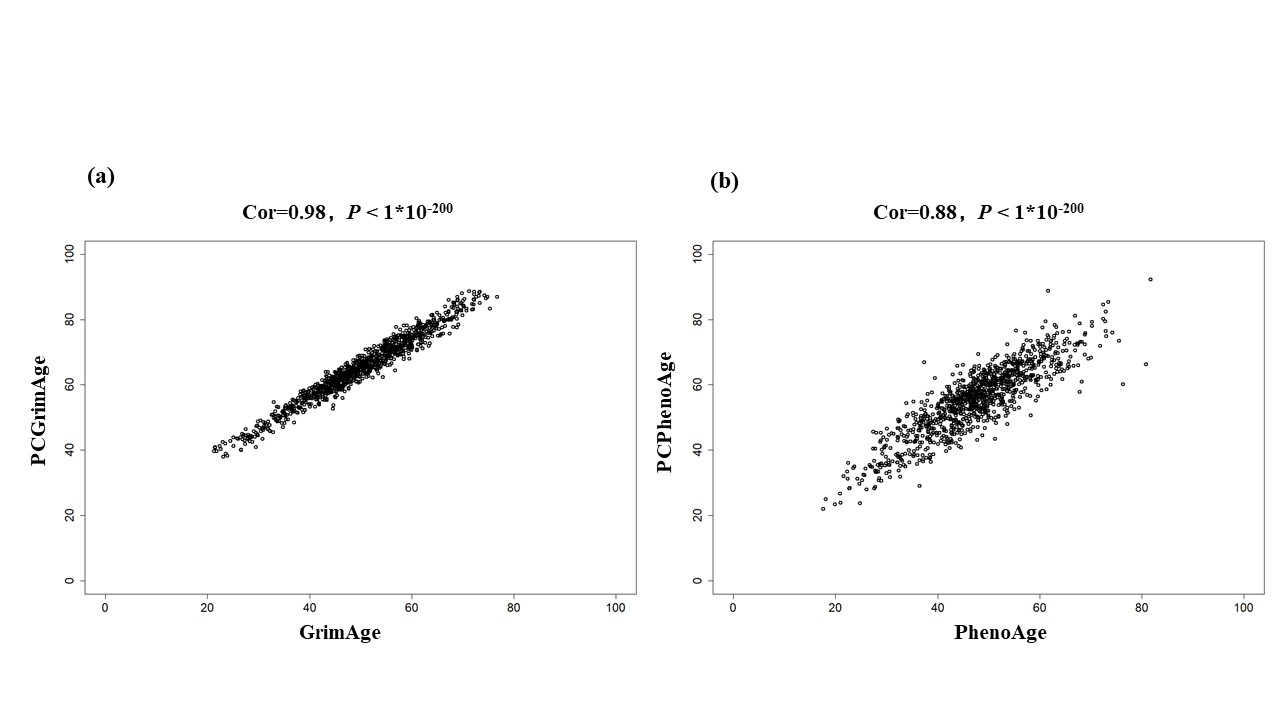


**Figure S1. Correlations between the original clocks and their PC clock proxies in the overall samples.**

Note: Correlation test *P* values based on Student’s t-distribution (two-tailed) are provided, without multiple-testing correction.


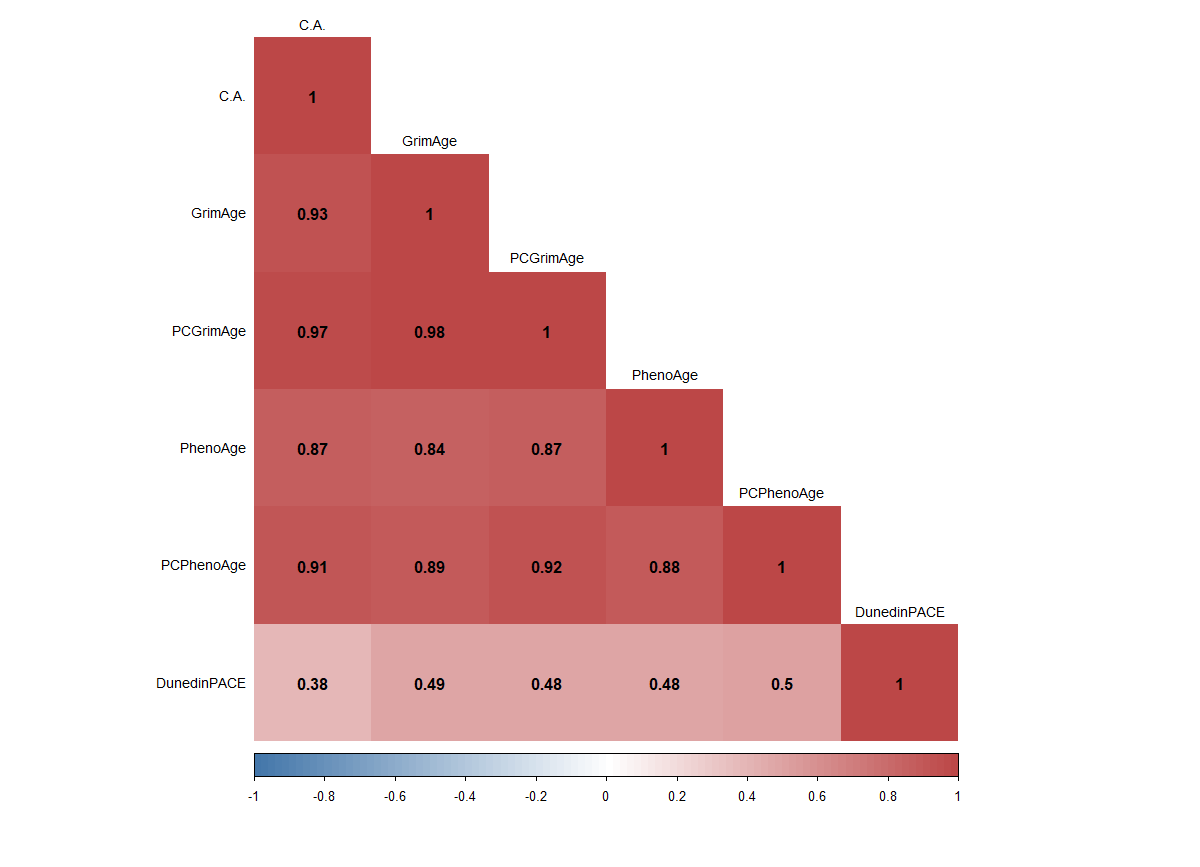


**Figure S2. Matrix heatmap of epigenetic age and chronological age**

Note: The number on the bottom left corner represents the Pearson correlation coefficient.

Abbreviations: C. A, chronological age
