# Supplementary material for: Five years of change in adult twins: longitudinal changes of genetic and environmental influence on epigenetic clocks
Source: BMC Med. 2024 Jul 10;22:289. doi: 10.1186/s12916-024-03511-y (PMC11234599; doi:10.1186/s12916-024-03511-y)
Supplement: Supplementary file 2 — Additional file 2: Table S1-S3. Table.S1- [Univariate model fit comparisons for the epigenetic age metrics in the cross-sectional analysis]. Table.S2- [Bivariate Model fit comparisons for the epigenetic age metrics in longitudinal analysis]. Table.S3- [Parameter estimates (95%CI) from bivariate twin models of epigenetic age metrics (N=268)]. [file 12916_2024_3511_MOESM2_ESM.docx]

**Table S1. Univariate model fit comparisons for the epigenetic age metrics in the cross-sectional analysis.**

| **Model** | **Compared to** | **AIC** | **-2LL** | **parameters** | **df** | **Δdf** | **Δ(-2LL)** | ***P*** |
| --- | --- | --- | --- | --- | --- | --- | --- | --- |
| **GrimAA** |  |  |  |  |  |  |  |  |
| Fully saturated | - | 2308.36 | 2286.36 | 11 | 943 | - | - | - |
| ACE | Fully saturated | 2300.24 | 2288.24 | 6 | 948 | 5 | 1.89 | 0.86 |
| ADE | Fully saturated | 2441.25 | 2431.25 | 5 | 949 | 6 | 144.89 | <0.01 |
| **AE** | **ACE** | **2299.30** | **2289.30** | **5** | **949** | **1** | **1.06** | **0.30** |
| CE | ACE | 2307.54 | 2297.54 | 5 | 949 | 1 | 9.30 | <0.01 |
| E | ACE | 2711.33 | 2705.33 | 3 | 951 | 3 | 417.09 | <0.01 |
| **PCGrimAA** |  |  |  |  |  |  |  |  |
| Fully saturated | - | 2194.97 | 2172.97 | 11 | 943 | - | - | - |
| ACE | Fully saturated | 2187.99 | 2175.99 | 6 | 948 | 5 | 3.02 | 0.70 |
| ADE | Fully saturated | 2349.27 | 2339.27 | 5 | 949 | 6 | 166.30 | <0.01 |
| **AE** | **ACE** | **2185.99** | **2175.99** | **5** | **949** | **1** | **<0.01** | **1.00** |
| CE | ACE | 2217.87 | 2207.87 | 5 | 949 | 1 | 31.88 | <0.01 |
| E | ACE | 2711.33 | 2705.33 | 3 | 951 | 3 | 529.34 | <0.01 |
| **PhenoAA** |  |  |  |  |  |  |  |  |
| Fully saturated | - | 2616.93 | 2594.93 | 11 | 943 | - | - | - |
| ACE | Fully saturated | 2613.37 | 2601.37 | 6 | 948 | 5 | 6.44 | 0.27 |
| ADE | Fully saturated | 2624.15 | 2614.15 | 5 | 949 | 6 | 19.22 | <0.01 |
| **AE** | **ACE** | **2611.94** | **2601.94** | **5** | **949** | **1** | **0.57** | **0.45** |
| CE | ACE | 2615.26 | 2605.26 | 5 | 949 | 1 | 3.89 | 0.05 |
| E | ACE | 2711.33 | 2705.33 | 3 | 951 | 3 | 103.96 | <0.01 |
| **PCPhenoAA** |  |  |  |  |  |  |  |  |
| Fully saturated | - | 2448.08 | 2426.08 | 11 | 943 | - | - | - |
| ACE | Fully saturated | 2440.75 | 2428.75 | 6 | 948 | 5 | 2.68 | 0.75 |
| ADE | Fully saturated | 2443.80 | 2433.80 | 5 | 949 | 6 | 7.72 | 0.26 |
| **AE** | **ACE** | **2439.60** | **2429.60** | **5** | **949** | **1** | **0.85** | **0.36** |
| CE | ACE | 2458.86 | 2448.86 | 5 | 949 | 1 | 20.11 | <0.01 |
| E | ACE | 2711.33 | 2705.33 | 3 | 951 | 3 | 276.58 | <0.01 |
| **DunedinPACE** |  |  |  |  |  |  |  |  |
| Fully saturated | - | 2415.42 | 2391.42 | 12 | 942 | - | - | - |
| **ACE** | **Fully saturated** | **2411.07** | **2397.07** | **7** | **947** | **5** | **5.66** | **0.34** |
| ADE | Fully saturated | 2516.04 | 2504.04 | 6 | 948 | 6 | 112.62 | <0.01 |
| AE | ACE | 2514.04 | 2504.04 | 5 | 949 | 2 | 106.97 | <0.01 |
| CE | ACE | 2425.43 | 2413.43 | 6 | 948 | 1 | 16.36 | <0.01 |
| E | ACE | 2579.94 | 2569.94 | 5 | 949 | 2 | 172.87 | <0.01 |

Note: A: additive genetic component; D: non-additive genetic component; C: shared environmental component; E: unique environmental component; Fully saturated: a baseline model of the observed data, which models the variances and means separately for each twin in a pair and across zygosity. -2LL: fit statistic, which is -2*log-likelihood of the data. df: degrees of freedom. AIC: Akaike’s Information Criteria, an alternative fit index. Lower values denote better model fits. Δχ2: difference in -2LL between two models, distributed chi-square. Δdf: difference in degrees of freedom between two models, which is equal to the difference in number of parameters. The best fitting models are marked as bold, selected based on the principle of parsimony and lowest AIC and -2ll value.

**Table S2. Bivariate Model fit comparisons for the epigenetic age metrics in longitudinal analysis.**

| **Model** | **Compared to** | **AIC** | **-2LL** | **parameters** | **df** | **Δdf** | **Δ(-2LL)** | ***P*** |
| --- | --- | --- | --- | --- | --- | --- | --- | --- |
| **GrimAA** |  |  |  |  |  |  |  |  |
| Fully saturated | - | 1317.32 | 1253.32 | 32 | 504 | - | - | - |
| ACE | Fully saturated | 1308.52 | 1278.52 | 15 | 521 | 17 | 25.20 | 0.09 |
| ADE | Fully saturated | 1311.53 | 1281.53 | 15 | 521 | 17 | 28.21 | 0.04 |
| **AE** | **ACE** | **1305.55** | **1281.55** | **12** | **524** | **3** | **3.02** | **0.39** |
| **PCGrimAA** |  |  |  |  |  |  |  |  |
| Fully saturated | - | 1146.93 | 1082.93 | 32 | 504 | - | - | - |
| ACE | Fully saturated | 1139.97 | 1109.97 | 15 | 521 | 17 | 27.05 | 0.06 |
| ADE | Fully saturated | 1140.68 | 1110.68 | 15 | 521 | 17 | 27.75 | 0.05 |
| **AE** | **ACE** | **1136.64** | **1112.64** | **12** | **524** | **3** | **2.67** | **0.45** |
| **PhenoAA** |  |  |  |  |  |  |  |  |
| Fully saturated | - | 1406.24 | 1342.24 | 32 | 504 | - | - | - |
| ACE | Fully saturated | 1413.39 | 1383.39 | 15 | 521 | 17 | 41.15 | <0.01 |
| ADE | Fully saturated | 1411.40 | 1381.40 | 15 | 521 | 17 | 39.17 | <0.01 |
| **AE** | **ADE** | **1407.39** | **1383.39** | **12** | **524** | **3** | **1.99** | **0.58** |
| **PCPhenoAA** |  |  |  |  |  |  |  |  |
| Fully saturated | - | 1234.34 | 1170.34 | 32 | 504 | - | - | - |
| ACE | Fully saturated | 1240.41 | 1210.41 | 15 | 521 | 17 | 40.07 | <0.01 |
| ADE | Fully saturated | 1247.62 | 1217.62 | 15 | 521 | 17 | 47.28 | <0.01 |
| **ACE** | **-** | **1240.41** | **1210.41** | **15** | **521** | **-** | **-** | **-** |
| AE | ACE | 1243.73 | 1219.73 | 12 | 524 | 3 | 9.32 | 0.03 |
| **DunedinPACE** |  |  |  |  |  |  |  |  |
| Fully saturated | - | 1273.63 | 1201.63 | 36 | 500 | - | - | - |
| ACE | Fully saturated | 1266.18 | 1228.18 | 19 | 517 | 17 | 26.54 | 0.07 |
| ADE | Fully saturated | 1265.73 | 1227.73 | 19 | 517 | 17 | 26.09 | 0.07 |
| **AE** | **ADE** | **1260.34** | **1228.34** | **16** | **520** | **3** | **0.62** | **0.89** |

Note: A: additive genetic component; D: non-additive genetic component; C: shared environmental component; E: unique environmental component; Fully saturated: a baseline model of the observed data, which models the variances and means separately for each twin in a pair and across zygosity. -2LL: fit statistic, which is -2*log-likelihood of the data. df: degrees of freedom. AIC: Akaike’s Information Criteria, an alternative fit index. Lower values denote better model fits. Δχ2: difference in -2LL between two models, distributed chi-square. Δdf: difference in degrees of freedom between two models, which is equal to the difference in number of parameters. The best fitting models are marked as bold, selected based on the principle of parsimony and lowest AIC and -2ll value.

**Table S3. Parameter estimates (95%CI) from bivariate twin models of epigenetic age metrics (N=268)**

| Epigenetic age metrics | Baseline | | | Follow-up | | |
| --- | --- | --- | --- | --- | --- | --- |
|  | h^2^ | c^2^/total variance | e^2^/ total variance | h^2^ | c^2^/total variance | e^2^/ total variance |
| GrimAA | 0.69(0.57,0.78) | - | 0.31(0.22,0.43) | 0.72(0.61,0.80) | - | 0.28(0.20,0.39) |
| PCGrimAA | 0.79(0.70,0.85) | - | 0.21(0.15,0.30) | 0.75(0.65,0.82) | - | 0.25(0.18,0.35) |
| PhenoAA | 0.52(0.34,0.66) | - | 0.48(0.34,0.66) | 0.57(0.40,0.70) | - | 0.43(0.30,0.60) |
| PCPhenoAA | 0.47(0.28,0.70) | 0.37(0.14,0.54) | 0.16(0.11,0.23) | 0.66(0.41,0.76) | 0.00(0.00,0.22) | 0.34(0.24,0.47) |
| DunedinPACE | 0.70(0.58,0.79) | - | 0.30(0.21,0.42) | 0.69(0.57,0.78) | - | 0.31(0.22,0.43) |

**Note:** h^2^: heritability, that is, the ratio of a^2^ to the total variance; a^2^, variance explained by additive genetic component; c^2^, variance explained by common environmental component; e^2^, variance explained by unique environmental component.
